# Comparative cytogenetics of two species of *Dermanura* (Chiroptera, Phyllostomidae) in Midwestern Brazil

**DOI:** 10.3897/CompCytogen.v15i2.60577

**Published:** 2021-04-02

**Authors:** Ricardo Firmino de Sousa, Paulo Cesar Venere, Karina de Cassia Faria

**Affiliations:** 1 Instituto de Ciências Biológicas, Programa de Pós-Graduação em Ecologia e Conservação da Biodiversidade, Universidade Federal do Mato Grosso – UFMT. Av. Fernando Corrêa da Costa, n° 2367, Bairro Boa Esperança. Cuiabá – MT, Brazil Universidade Federal do Mato Grosso Cuiabá Brazil; 2 Faculdade de Ciências Agrárias, Biológicas e Sociais Aplicadas, Universidade do Estado de Mato Grosso, Av. Prof. Dr. Renato Figueiro Varella, s/n, Nova Xavantina, MT, Brazil Universidade Federal do Mato Grosso Cuiabá Brazil

**Keywords:** Bats, Stenodermatinae, *Dermanura gnoma*, *Dermanura anderseni*, karyotype

## Abstract

*Dermanura* Gervais, 1856 is represented by small frugivorous bats of the Stenodermatinae subfamily. The taxonomy of this group presents controversies and has been subject to changes, especially since the morphological characters evaluated have left gaps that are difficult to fill regarding good species characterization. Previous studies performed in *Dermanura cinerea* Gervais, 1856 found that the karyotype of this species has a diploid number of chromosomes equal to 30 and 56 autosomal arms. The objective of the present study was to describe, for the first time, the karyotypes of the species *Dermanura anderseni* (Osgood, 1916) and *Dermanura gnoma* (Handley, 1987) based on classical cytogenetic markers. For both species, the diploid number found was 2n = 30 and NFa = 56. Two pairs of chromosomes showed markings of the nucleolus organizing regions (AgNORs) in the species *D. anderseni* and only one pair in *D. gnoma*, differing from what has already been described for *D. cinerea*. The two species analyzed here also showed differences in the sex chromosome system, with *D. gnoma* showing a neo-XY type system while in *D. anderseni* the classic XY sexual system was observed. In both species, visualization of the constitutive heterochromatin occurred in the pericentromeric region of all chromosomes, as well as in the short arms of the subtelocentric chromosomes. The present work represents an important expansion of karyotypic information for the subfamily Stenodermatinae, bringing chromosomal features that are possible to use in the taxonomic implications of the group.

## Introduction

Among bat families, Phyllostomidae Gray, 1825 is the most morphologically and ecologically diverse, with more than 200 species arranged in 60 genera and 11 subfamilies (Solari and Martinez-Arias 2014; Baker et al. 2016). The subfamily Stenodermatinae Gervais, 1856 is represented in Brazil by 13 genera and 35 species (Nogueira et al. 2014; Pereira et al. 2017). Some genera of this subfamily are complex regarding their phylogenetic relationship, as is the case of *Artibeus* Leach, 1821, for which several questions have been raised (Van-Den-Bussche et al. 1993; Hoofer et al. 2008). Owen (1991) divided this genus into three taxa based on morphological characters: *Artibeus* Leach, 1821 (species with large body), *Dermanura* Gervais, 1856 (species with small body) and *Koopmania* Owen, 1991 (with one representative: *Artibeus concolor* Peters, 1865, with intermediate body size). Through analyses of the mitochondrial gene cytochrome-b and satellite DNA sequences, Van-Den-Bussche et al. (1998) suggested that *Artibeus* and *Dermanura* are sister groups.

The genus *Dermanura* has 11 described species (Hoofer et al. 2008; Solari et al. 2009). Of these, four occur in Brazil: *Dermanura anderseni* (Osgood, 1916), *Dermanura bogotensis* (Andersen, 1906), *Dermanura cinerea* (Gervais, 1856) and *Dermanura gnoma* (Handley, 1987) (Nogueira et al. 2014). The taxonomy of this group presents controversies and has been subject to alterations based on the character set evaluated (Redondo et al. 2008). In general, species identification has been based on morphological and metric characteristics (skull, body size, forearm and weight measurements), which makes taxonomic identification and the establishment of phylogenetic relationships difficult (Sotero-Caio et al. 2017a).

With the advancement in molecular techniques, and cytogenetic approaches, the integrative analysis becomes important, since more robust proposals are provided to identify the group’s diversification and evolution patterns (Sotero-Caio et al. 2015; Tavares et al. 2015; Bassantes et al. 2020). Comparative karyotypic studies in bats, especially within Phyllostomidae, have indicated great examples of species with a high level of chromosomal reorganization, diverging from the ancestral species (Pieczarka et al. 2013; Sotero-Caio et al. 2017a, b).

However, knowledge about karyotype data is still scarce for some bat species, and little data has been published for species from South America. Recent studies have been complementing data on chromosomal banding for some species (Calixto et al. 2014; Corrêa and Bonvicino 2016; Sousa et al. 2017; Souza et al. 2017; Côrrea et al. 2017), with karyotype descriptions for species that do not present chromosomal data in the literature (Santos et al. 2002; Garcia and Pessôa 2010; Almeida et al. 2016, Gomes et al. 2016).

Even for many species of Chiroptera, which currently have a reasonable amount of karyotype data, some important gaps can be detected through a detailed data survey. These gaps highlight the importance of expanding chromosomal studies with bats in order to make the information available for the order more robust, especially regarding knowledge about Chiroptera’s chromosomal evolution (Marchesin and Morielle-Versute 2004; Garcia and Pessôa 2010; Sotero-Caio et al. 2017a).

In *Dermanura*, the only chromosome set ever described is for *D. cinerea* whose analyses revealed a diploid number of chromosomes equal to 30 and 56 autosomal arms (Santos and Souza 1998b; Noronha 2010). The present work aims to describe the karyotypes of the species *Dermanura anderseni* and *Dermanura gnoma* based on classic cytogenetic markers (conventional staining with *Giemsa*, C-banding and silver nitrate impregnation) in order to identify chromosomal morphology of these species and contribute to the cytogenetic knowledge of bat species.

## Material and methods

**Figure 1.**
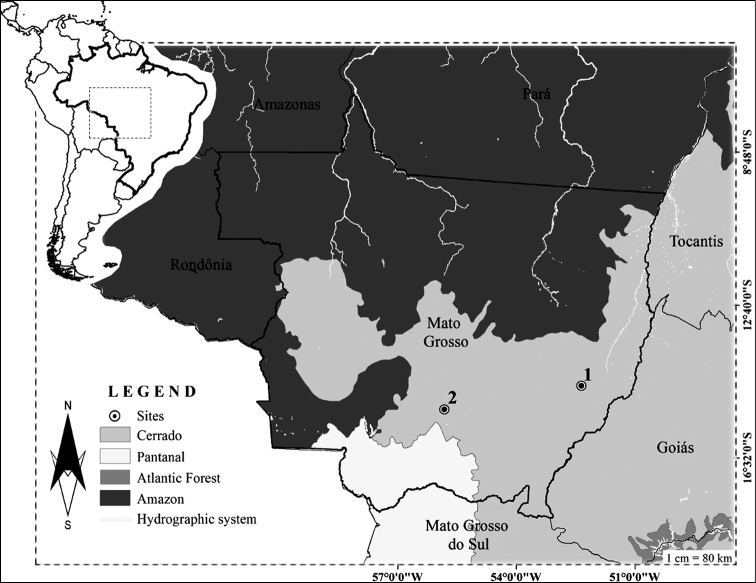
Capture locations of the species *Dermanura anderseni* and *Dermanura gnoma***1** Nova Xavantina, in Cerradão and Cerrado *strictosensu* areas and **2** Chapada dos Guimarães in a Cerrado *strictosensu* area, Mato Grosso, Brazil.

Specimens were collected in the municipalities of Nova Xavantina (14°42'28.8"S, 52°21'03.9"W) and Chapada dos Guimarães (15°18'25.57"S, 55°49'06.33"W), both in the state of Mato Grosso – Brazil (Fig. 1), which presented two Cerrado Biome phytophysiognomies: (I) Cerradão, a forest type in the Central Brazilian Plateau, with close treetops and a plant community that presents a closed appearance, but with spacing between trees that allows sun to penetrate through and tree heights ranging from 10 to 15 m (Rizzini 1997). (II) Cerrado *strictosensu*, which is composed of more spaced out, smaller, and twisted trees, providing the appearance of grasses and subshrubs with low vegetation prevailing (Rizzini 1997). Trees in these areas are usually 3 to 6 m high and can reach up to 10 m (Ribeiro and Walter 2008). Five specimens of *Dermanura anderseni* Osgood, 1916 were analyzed, three (2 females and 1 male) captured in the municipality of Nova Xavantina and two (1 male and 1 female) captured in Chapada dos Guimarães. For *Dermanura gnoma* Handley, 1987, two male specimens captured in Nova Xavantina were analyzed. Bats were captured with mist nets (9 × 3 m, with 16 mm mesh) set up from 18:00h to 00:00h in possible bat routes, which were inspected every 30 minutes. The animals were recorded and identified to species level following specialized bibliography (Vizotto and Taddei 1973; Gardner 2008; Reis et al. 2013; Nogueira et al. 2014; Reis et al. 2017). Both species have very evident white facial stripes, however, among other differences, they have different sizes (with forearm 38 to 40 mm in *D. anderseni* and 34 to 38 mm in *D. gnoma*) and different dental formulas (I2 / 2, C1 / 1, P2 / 2, M2 / 2 = 32 for *D. anderseni* and I2 / 2, C1 / 1, P2 / 2, M2 / 3 = 30 for *D. gnoma*). Specimens captured in Nova Xavantina were deposited in the scientific collection of the Laboratório de Genética at the Universidade do Estado de Mato Grosso Nova Xavantina campus under the following collection numbers: RM 216, 234, 302 (*D. anderseni*) and 257, 333 (*D. gnoma*) (License No. 18276-1 – IBAMA/SISBIO/MT) and specimens captured in Chapada dos Guimarães were deposited in the zoological collection of the Instituto de Biociências at the Universidade Federal de Mato Grosso, Cuiabá campus, under license no. 46359-1 – ICMBio/SISBIO/MT (UFMT-PNCG 863 and 866, *D. anderseni*).

Chromosomal preparations were obtained through direct bone marrow extraction, according to the procedure described in Morielle-Versute et al. (1996), with minor adjustments during routine work in the laboratory. To observe the metaphases, conventional staining with Giemsa was performed. The technique for visualizing constitutive heterochromatin (C-banding) was performed according to the protocol proposed by Sumner (1972); staining with propidium iodide and silver nitrate impregnation (AgNORs) followed Howell and Black (1980).

The slides with chromosomal preparations were analyzed using optical microscopy. Slides that showed good quality metaphases were photographed under an Olympus BX51 microscope (Tokyo, Japan). The free edition of Adobe Photoshop Cs6 portable program was used to assemble karyotypes. Afterwards, chromosomes were measured and classified according to the position of the centromere, following Levan et al. (1964).

## Results

**Figure 2.**
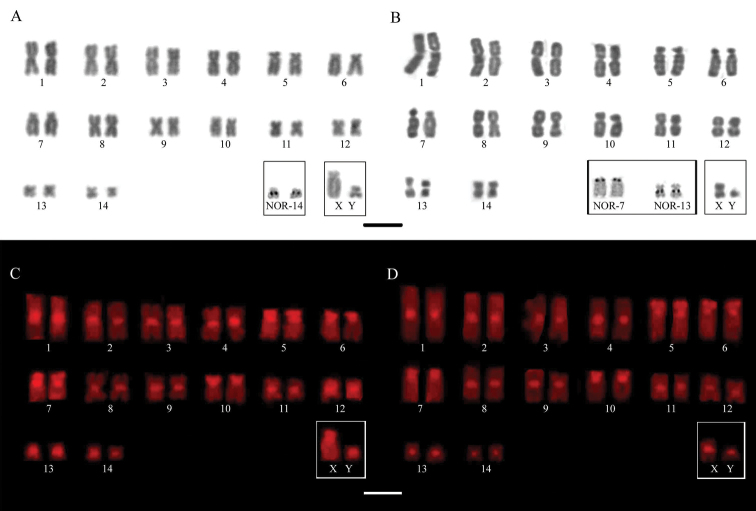
**A, B** karyotypes of male individuals stained with *Giemsa*, highlighted in the boxes the AgNORs marks and the pair of sex chromosomes of **A***Dermanura gnoma* (2n = 30 and NFa= 56) and **B***Dermanura anderseni* (2n = 30 and NFa = 56) **C, D** karyotypes showing the distribution of constitutive heterochromatin for both species studied **C***D. gnoma* and **D***D. anderseni*. Scale bar: 5 μm.

The diploid number found for *Dermanura gnoma* Handley, 1987 was 2n = 30 and the number of autosomal chromosome arms was NFa = 56. The karyotypes are composed of ten pairs of meta-submetacentric chromosomes (1, 2, 3, 4, 8, 9, 11, 12, 13 and 14) and four subtelocentric chromosome pairs (5, 6, 7 and 10) (Fig. 2A). The X chromosome is a large subtelocentric and the Y chromosome is a small metacentric. With silver nitrate staining, the nucleolus organizing regions (AgNORs) were observed only in the number 14 pair, in the interstitial position of the long arm (Fig. 2A). In this species, C-banding showed constitutive heterochromatin markings in the pericentromeric regions of all autosomal chromosomes and in X and Y sex chromosomes. Small blocks of heterochromatin were also observed in interstitial regions in the short arms of pairs 5, 6, 7, 10 and on chromosome X (Fig. 2C).

The karyotype of the species *Dermanura anderseni* Osgood, 1916 also presented the diploid number 2n = 30 and the number of autosomal chromosome arms NFa = 56. Similar to *D. gnoma*, the karyotype of this species also presents ten pairs of metacentric chromosomes (1, 2, 3, 4, 8, 9, 11, 12, 13 and 14) and four pairs of subtelocentric chromosomes (5, 6, 7 and 10). The X chromosome is a medium metacentric and the Y chromosome is a small acrocentric. AgNORs were observed in two chromosome pairs: in the short arm of subtelocentric pair no. 7 and in the interstitial position of the long arm of chromosome 13 (Fig. 2B). The constitutive heterochromatin regions presented pericentromeric markings on all autosomal chromosomes and on the X and Y sex chromosomes, in addition to small blocks on the short arms of the subtelocentric pairs 5, 6, 7 and 10 (Fig. 2D).

## Discussion

The cytogenetic analyses presented here indicated that for both species, *D. anderseni* and *D. gnoma*, the diploid number found was 2n = 30 and NFa = 56. Among the four species of *Dermanura* that occur in Brazil, only the *D. cinerea* karyotype was previously known, with karyotype form also described as 2n = 30 and NFa = 56 (Tucker 1986; Souza and Araújo 1990; Santos and Souza 1998b; Santos et al. 2002; Calixto et al. 2014; Sotero-Caio et al. 2017b). However, the analysis of the band patterns showed relevant differences on the C-banding, AgNORs patterns, and on chromosome morphologies of the sexual pair between these species.

Our results verified that the sexual system described as neo-XY was found in *D. gnoma*, while in *D. anderseni* the classic XY sexual system was observed, where X is a medium metacentric and Y is a small acrocentric. The classic XY sexual system is the most common for bat species studied to date (Baker 1970; Varella-Garcia et al. 1989; Pieczarka et al. 2005; Calixto et al. 2014). According to Tucker’s proposal (1986), the neo-XY sexual system had its origin when the short arm of the X chromosome underwent fission and there was fusion with the Y chromosome, forming a small metacentric Y and the X chromosome is represented by a large subtelocentric. This system was also observed by Souza and Araújo (1990) in *D. cinerea*, suggesting that this condition is apomorphic in relation to the condition observed in *D. anderseni*.

Regarding the location of the ribosomal sites, Santos et al. (2002) described multiple AgNORs for *D. cinerea* involving pairs 10 (medium metacentric) and 13 (small metacentric). In the present study, multiple AgNORs were observed for *D. anderseni*, however, involving pairs 7 (medium subtelocentric) and 13 (small metacentric). In *D. gnoma* the AgNORs are localized in a single and different pair of chromosomes (pair 14 – small metacentric). Thus, this marker is relatively well informative for this group of bats and can, if used with caution, be a good species-specific indicator.

Observations of the nucleolus organizing regions can be performed through FISH (fluorescence in situ hybridization) using probes for rDNA 5S, 18S and 45S (Calixto et al. 2014). In the genus *Dermanura*, FISH for rDNA 18S were only observed in *D. cinerea* in pairs 9, 10 and 13 (Santos et al. 2002), confirming the occurrence of a multiple system of rDNA sites. However, this technique has not yet been applied to other species within the genus.

Studies on the distribution pattern of constitutive heterochromatins (C+ bands) agreed with what is generally observed among bats; that is, the presence of blocks of C+ bands in the pericentromeric regions of all chromosomes and in the terminal regions of the subtelocentric chromosome pairs (Souza and Araújo 1990; Santos and Souza 1998b; Rodrigues et al. 2003; Silva et al. 2005; Garcia and Pessôa 2010; Lemos-Pinto et al. 2012; Calixto et al. 2014).

For the family Phyllostomidae, the C+ bands are located in the pericentromeric regions of the chromosomes (Varella-Garcia et al. 1989; Rodrigues et al. 2000; Barros et al. 2009; Sbragia et al. 2010); however, additional conspicuous heterochromatic blocks have been found in interstitial and telomeric regions in several species, such as *Carollia perspicillata* (Linnaeus, 1758), *Choeroniscus minor* (Peters, 1868), *Glossophaga soricina* (Pallas, 1766), *Artibeus lituratus* (Olfers, 1818), *A. planirostris* (Spix, 1823), *Dermanura cinerea* (Gervais, 1856), *Sturnira lilium* (É. Geoffroy, 1810), *Platyrrhinus lineatus* (É. Geoffroy, 1810), *Uroderma magnirostrum* Davis, 1968, *U. bilobatum* Peters, 1866, *Diaemus youngi* (Jentnik, 1893), *Desmodus rotundus* (É. Geoffroy, 1810) and *Diphylla ecaudata* (Spix, 1823) (Varella-Garcia et al. 1989; Souza and Araújo 1990; Santos and Souza 1998a, b; Neves et al. 2001; Santos et al. 2001; Silva et al. 2005). The constitutive heterochromatin observed in the chromosomes of *D. gnoma* and *D. anderseni* follows a distribution pattern similar to that found in bats of the Phyllostomidae family.

Studies conducted with chromosomal mapping of different bat families have revealed that this group is characterized by karyotypic conservation, caused by slow chromosomal evolution (Baker et al. 1980; Varella-Garcia et al. 1989; Souza and Araújo 1990; Morielle-Versute et al. 1996; Pieczarka et al. 2005). However, in a general analysis of the chromosomal data available for the different species already studied, it seems that this conservatism is not a rule. In Phyllostomidae, several subfamilies seem to have relatively divergent karyotypes, at least in their microstructure. In addition, there is a diversity of karyotypic formulas that are quite interesting when comparing different subfamily groups, which supports karyotype studies as an important study area (Pieczarka et al. 2013; Ribas et al. 2015; Sotero-Caio et al. 2015, 2017a).

Overall, the data presented herein expands the cytogenetic knowledge of bats in the Stenodermatinae subfamily and is the first work to analyze the karyotypes of *Dermanura gnoma* and *D. anderseni*. The comparative analysis of the species of this subfamily reveals high conservation for this group and reinforces its position as a well-established phylogenetic unit within the order Chiroptera.
